# Correspondence: Reply to ‘Challenging a proposed role for TRPC5 in aortic baroreceptor pressure-sensing’

**DOI:** 10.1038/s41467-017-02704-9

**Published:** 2018-03-23

**Authors:** On-Chai Lau, Bing Shen, Ching-On Wong, Xiaoqiang Yao

**Affiliations:** 1 Institute of Neuroscience, University of Louvain, Brussels, B1200 Belgium; 2 Department of Physiology, Anhui Medical University, Hefei, 230032 China; 3 Department of Integrative Biology and Pharmacology, McGovern Medical University, The University of Texas Health Science Centre, Houston, TX 77030 USA; 4 School of Biomedical Sciences, Chinese University of Hong Kong, Hong Kong, China

## Introduction

In our recent paper^1^, we used a variety of in vitro and in vivo methods to demonstrate participation of TRPC5 in mechanosensation in aortic baroreceptors. The correspondence by Thakore et al. raised a number of concerns about our findings^2^.

We originally reported that the mean arterial pressure in *Trpc5*^*−/−*^ mice was higher than in wild-type mice, which differs from observations made by Thakore et al.^2^ Upon re-examination of our original data we identified two outliers with unusually high blood pressure readings, which we assume result from a technical issue that arose during the measurements. Removal of these two data sets resulted in no statistically significant difference in the resting mean arterial pressure between *Trpc5*^*−/−*^ and wild-type mice (123 ± 8 mmHg, *n* = 10 vs. 106 ± 5 mmHg, *n* = 10 (*P* > 0.05) by Student’s *t*-test). In addition, we performed blood pressure measurements in two more pairs of mice after the paper’s publication. After combining these measurements, the summarized values for the resting mean arterial blood pressure are: *Trpc5*^*−/−*^ mice 121 ± 8 mmHg, *n* = 12 vs. wild-type mice 105 ± 4 mmHg, *n* = 12, (*P* > 0.05) by Student’s *t*-test. These errors are also described in an Author Correction associated with our original paper, and raw data for all resting mean arterial blood pressure measurements for the two mouse cohorts are presented as Supplementary Data 1 associated with the correction. The replacement of these two data points does not however affect our main conclusion, namely that the minute-by-minute variation of blood pressure was larger in *Trpc5*^*−/−*^ mice than in wild-type mice, as a statistically significant difference in range of blood pressure variation over 24 h remains (60 ± 6 mmHg, *n* = 12 for normal mice and 114 ± 22 mmHg, *n = *12 for *Trpc5*^*−/−*^ mice, *P* < 0.05 by Student’s *t*-test).

Thakore et al. also questioned whether the change in our conclusion regarding the mean arterial pressure would affect our conclusion that TRPC5 participates in mechanosensation in aortic baroreceptors. We want to emphasize that the correction of the resting mean arterial pressure values in *Trpc5*^*−/−*^ mice does not affect this conclusion. While blood pressure fluctuation is a manifestation of baroreceptor dysfunction, mean arterial pressure is not. It is well recognized that baroreceptor dysfunction does not alter long-term mean arterial pressure. Seminal studies by Cowley and colleagues^3^ showed that, after denervation of baroreceptor nerves in dogs, the long-term mean arterial pressure was not altered. The reported average blood pressures were 107 mmHg (*n = *41) compared to 105 mmHg (*n = *40) measured in intact dogs under similar conditions^4^. Their findings about no alteration of mean arterial pressure after baroreceptor denervation were later confirmed in rats^5^, rabbits^6^, and monkeys^7^. The reason why baroreceptor dysfunction does not alter long-term arterial pressure is that baroreceptors quickly adapt and reset themselves^8^. Therefore, the erroneously calculated difference in mean arterial pressure between the *Trp5*^*−/−*^ and wild type mice described in the original paper was never used as a supporting point for our conclusion of TRPC5 involvement in baroreceptor mechanosensing.

**Fig. 1:**
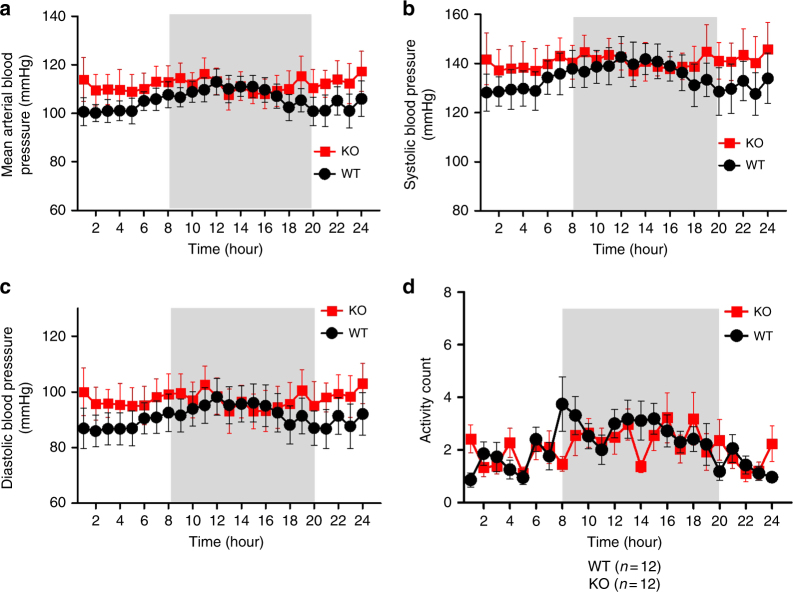
Cardiovascular and behavioral activities in freely moving WT mice and *Trpc5*^*−/−*^ mice. Shown are 24 h recording of mean arterial pressure (**a**), systolic pressure (**b**), diastolic pressure (**c**) and activity count (**d**). The recordings were performed from 1000 hours to 1000 hours of the next day. Shaded areas represent dark period when the light was switched off (1800 hours to 0600 hours of the next day). WT, wild-type mice; KO, *Trpc5*^*−/−*^ mice; *n* = 12

**Fig. 2:**
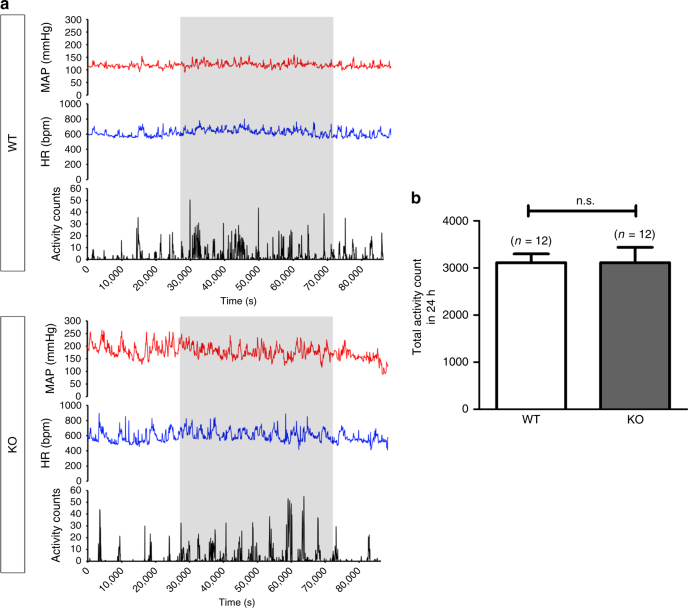
Freely moving conscious wild-type and *Trpc5*^*−/−*^ mice show similar locomotion activity. **a** Representative traces for 24 h recording of mean blood pressure, heart rate and locomotion activity count in 1 min time interval in a wild-type mouse (upper) and a *Trpc5*^*−/−*^ (lower) mouse measured by telemetry. Shaded regions represent the night time from 1800 hours to 0600 hours of the next day, in which the animals were more active. **b** Summarized data showing the total activity count in 24 h. Mean ± s.e.m. *n = *12. n.s. (not significant, *P* > 0.05). Statistical analysis was performed by Student’s *t*-test

It was not our original intention to study diurnal variation in our publication^1^. However, at the suggestion of Thakore et al., we analyzed the diurnal change of the mean arterial pressure and associated activity count from multiple mice. Our data agree with those from Thakore et al., showing no statistically significant difference between *Trpc5*^*−/−*^ and wild-type mice with regard to their mean arterial pressure, hourly diurnal change in arterial pressure and activity count (Fig. 1). Nevertheless, please note that these parameters are not related to baroreceptor function. For the purpose of studying the baroreceptor-related blood pressure stability, it is necessary to examine the blood pressure variations in time intervals that are a minute or less long. When hourly blood pressure values are averaged, the information about blood pressure stability is lost. In addition, Thakore et al. asked why the activity measurements from the DSI system were not correlated with the blood pressure values^2^. In the published article^1^, we opted to measure the activity count using the open field test, following the advice of experts in neuroscience and the DSI technique who recommended the open field test as the optimal approach for assessing the animal locomotion. In response to the comments of Thakore et al., we have now analysed the activity count data from telemetric devices (Fig. 2). As expected, the new data are in complete agreement with the results gathered from the open field test.

Another concern raised by Thakore et al. is related to electrophysiological recordings and molecular compositions (homomeric vs. heteromeric) of TRPC5-containing channels in nodose baroreceptor neurons^2^. In our published study, we did not elaborate the molecular composition of TRPC5-containing channels in nodose ganglion neurons (homomeric or heteromeric or a mixture)^1^. Native nodose ganglion neurons contain multiple cation channels with different conductance, making it difficult to differentiate between those that contain TRPC5 and those that do not. Therefore, in single channel recordings and some whole-cell experiments, 20 μM La^3+^ was included to inhibit other cation channels^9^. The inclusion of La^3+^ greatly accelerated the progress of our electrophysiological studies. Thakore et al. suggested that the recorded single channels are homomeric TRPC5 channels, because the single channel conductance in the presence of La^3+^ is 25 pS. We agree with this point. We also agree with their viewpoint that the whole-cell *I–V* curve, especially in the absence of La^3+^, does not have a homomeric-TRPC5-like double-rectifying shape. We believe that this discrepancy in the shape of the whole-cell *I–V* curve could be due to the additional contribution of other mechanosensitive components (other than TRPC5) and/or the additional contribution of heteromeric TRPC5 channels, and these possibilities are also mentioned by Thakore et al. In our publication, we recognized and acknowledged the possible contribution of other mechanosensitive components such as ASICII and TRPV1. TRPC5 is known to co-assemble with TRPC1, TRPC4, TRPC1-C4, TRPC1-C3^10,11^. The reported shapes of whole-cell *I–V* curves for these heteromeric TRPC5 channels are either outwardly-rectifying^10,11^ or nearly linear^12^. The single channel conductance of heteromeric TRPC1-TRPC5 channels is much smaller, with a reported value of ~5 pS in the absence of La^3+^^10^. In the presence La^3+^, the single channel conductance of heteromeric TRPC1-TRPC5 should presumably be even smaller^9^ and therefore difficult to resolve. It is likely that we may have missed out these very small channels as “background noise” in our single channel recording. Taken together, native baroreceptor neurons may contain a mixture of mechanosensitive channels, including homomeric TRPC5, heteromeric TRPC5 and other cation channels. This could explain why the whole-cell *I–V* curve did not resemble the double-rectifying shape typical of homomeric TRPC5 presented in Figs. 2 and 6 of our original paper. On the other hand, in single channel recording, inclusion of La^3+^ may only allow for recording of homomeric TRPC5 channels. However, overall we feel that there is much speculation in the above discussion. Currently, we do not have sufficient experimental data defining the molecular compositions of these TRPC5-containing channels in nodose baroreceptor neurons. Further experiments using advanced molecular and biochemical methods in combination with electrophysiology are needed to accurately resolve the molecular compositions of these TRPC5-containing channels. At the present, we would rather name them as TRPC5-containing channels. In our paper, we have stated that “further studies are still needed to determine the molecular composition of TRPC5-containing channels in aortic baroreceptor neurons”.

**Fig. 3:**
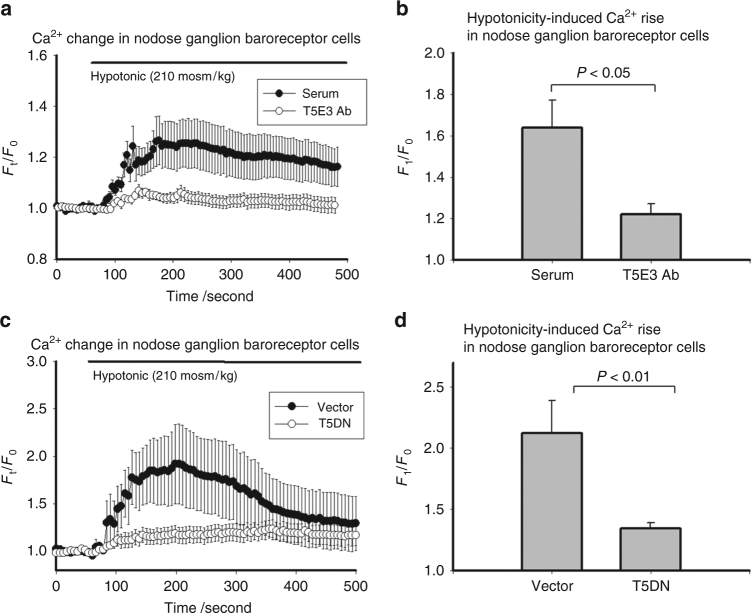
Hypotonicity-induced [Ca^2+^]_i_ rise in rat baroreceptor neurons is inhibited by T5E3 and T5DN. **a** and **c** Representative time-series graphs for [Ca^2+^]_i_ fluorescent signal in primary cultured DiI-positive neurons from rat nodose ganglia in response to hypotonicity (210 mOsm). The data points represent the mean ± s.e.m. for ≥7 cells in a representative experiment (*n* = 6). **b** and **d** Summarized mean ± s.e.m. (*n* = 6 separate experiments) data for the maximal [Ca^2+^]_i_ increase (F_1_/F_0_) in response to hypotonicity (210 mOsm) as in **a** and **c**, respectively. In **a** and **b**, cells were pre-incubated with a TRPC5-blocking antibody T5E3 (4 μg ml^−1^) or pre-immune IgG (4 μg ml^−1^, labeled as “pre-immune”) for 15 min. In **c** and **d** cells were transfected with 5 µg dominant-negative TRPC5 (T5DN) or pCDNA6c (vector) by electroporation

While Thakore et al. agree that the recorded single channel activity derives from TRPC5-containing channels, they questioned the importance of TRPC5-containing channels in mechanosensitivity in the absence of La^3+^. They further speculated that TRPC5 may not be sufficiently activated in the absence of La^3+^ to significantly alter baroreceptor function. We strongly disagree with these speculations. It is evident that the whole-cell current in patch clamp can largely be attributed to TRPC5, because the hypotonicity- and pressure-activated current was markedly reduced by T5E3, T5DN and TRPC5 knockout with or without La^3+^ (Figs. 2 and 6 of the original paper). Furthermore, siRNA-mediated TRPC5 knockdown markedly reduced the pressure-activated whole-cell current in the absence of La^3+^ (Supplementary Fig. 2 of the original paper). In the same vein, we now provide another piece of evidence which shows the hypotonicity-activated Ca^2+^ rise mediated by TRPC5 in nodose baroreceptor neurons in the absence of La^3+^ (Fig. 3). In Fig. 3, hypotonicity-induced [Ca^2+^]_i_ rise in the absence of La^3+^ in rat baroreceptor neurons was found to be inhibited by an anti-TRPC5 blocking antibody T5E3 and a dominant negative construct T5DN. Importantly, our in vivo experiments provide more direct evidence. We have shown the impairment of pressure-dependent action potential firings in rats expressing the dominant-negative variant of TRPC5 and *Trpc5*^*−/−*^ mice (Figs. 4 and 7 of the original paper). Another commonly-used index of baroreceptor function is the reflex control of heart rate when blood pressure is acutely altered. Our data clearly show a deficiency in baroreflex control of heart rate in TRPC5-disrupted rats and *Trpc5*^*−/−*^ mice (Fig. 9 and Supplementary Fig. 4 of the original paper). Our notion was further supported by unstable minute-to-minute blood pressure variation in *Trpc5*^*−/−*^ mice (Fig. 8 of the original paper). Note that the aforementioned in vitro Ca^2+^ experiments and all in vivo experiments were performed in the absence of La^3+^.

Lastly, Thakore et al. questioned the relevance of hypotonicity and hydrostatic pressure, which we used in patch clamp recordings, to the mechanical force experienced by baroreceptors in vivo^2^. We fully agree that these are indeed the limitation of our in vitro experiments. We believe that the best approach should be in vivo animal experiments examining the pressure-induced action potential firings, as well as baroreflex control of heart rate, which we have shown in Figs. 4, 7, 9 and Supplementary Fig. 4 of the published paper. A minor point in the Correspondence by Thakore et al. is that in the original Fig. 6h, the pressure-activated whole-cell current in baroreceptor neurons at −80 mV is not statistically different between wild-type and *Trpc5*^*−/−*^ mice. We hope that it is understandable that the relatively small current amplitude at negative voltage made it more difficult to resolve the difference. On the other hand, it is clear that the current amplitude at positive voltage is reduced in baroreceptor neurons from *Trpc5*^*−/−*^ mice compared to those from wild-type mice (original Fig. 6h). Furthermore, T5E3 inhibited the pressure-activated currents at both positive and negative voltages in baroreceptor neurons from wild-type mice (original Fig. 6g).

We truly appreciate the valuable comments from Dr. Susan Brain in our email communications as well as the correspondence from Thakore et al., as their insightful criticism has prompted us to re-examine and correct our data related to the difference in the mean arterial pressure between *Trpc5*^*−/−*^ and wild-type mice. However, this correction in mean arterial pressure does not affect our main conclusion that TRPC5 is involved in baroreceptor mechanosensing. We are also grateful to Dr. David Beech for his thoughtful comments on electrophysiology and suggestions for further experiments. We acknowledge that further experiments are needed to clarify the molecular compositions of TRPC5-containing channels in native baroreceptor neurons. However, we believe that we have provided sufficient data, collected from both in vitro and in vivo experiments, to demonstrate that TRPC5-containinng channels play a role in baroreceptor mechanosensing.

## Methods

### Telemetric recording of blood pressure

The method was modified from a previous protocol^13^. Anaesthesia of 129S1/SvImJ mice was induced and maintained with 3 and 1.5% isoflurane, in a 3:1 O_2_ to air ratio, respectively. The catheter was inserted into the right common carotid artery and advanced in order to place the blood pressure sensor in aorta for blood pressure measurement. The telemetric device, HD-X11, was embedded around the abdominal region subcutaneously. The animals were allowed to recover for at least a week before recording. Twenty-four hour telemetric recording of blood pressure and heart rate were performed in the conscious animals with DSI Dataquest A.R.T. telemetry system (Data Science International). The data were analysed using Spike2 (version 6.06, Cambridge Electronic Design). Average mean arterial blood pressure and heart rate in 1 min time interval were plotted continuously for 24 h from 1000 hours to 1000 hours of the next day. The mean arterial blood pressure was calculated based on the standard equation of MAP = (1/3 × SBP) + (2/3 × DBP). Frequency distribution histogram of the mean arterial blood pressure recorded in a 24-h period was plotted with the bandwidth of 15 mm Hg pressure range. The highest and lowest 2.5% of the recorded blood pressure values were discarded in order to remove possible noise. After that, the range of blood pressure variation was obtained by subtracting the lowest leftover value from the highest leftover value of the mean arterial pressure.

### [Ca^2+^]_i_ measurement

Nodose ganglion neurons were isolated and cultured from Sprague–Dawley rats as described^1^. The concentration of intracellular Ca^2+^ ([Ca^2+^]_i_) was measured, as described elsewhere^14^. Briefly, culture neurons were seeded onto poly-L-lysine-coated glass discs. The cells were loaded for 45 min in dark with 5 μM Fluo-4/AM and 0.02% pluronic F-127 in culture media, before mounting onto a microscope chamber containing an isotonic bath solution (in mM: 65 NaCl, 5 KCl, 1 CaCl_2_, 1 MgCl_2_, 10 HEPES, pH 7.4; osmolarity calibrated to 300 mOsm with ~140 mM mannitol). Hypotonic bath solutions contained identical ionic concentrations to isotonic bath solution, and the osmolarity calibrated to different values by varying the concentration of mannitol. Normal physiological saline solution (NPSS) contained in mM: 140 NaCl, 5 KCl, 2 MgCl_2_, 1 CaCl_2_, 10 Glucose, 10 HEPES, pH 7.4. Fluorescence [Ca^2+^]_i_ signals were recorded by InCytIm Basic live cell imaging system (Intracellular Imaging Inc. USA). Changes in [Ca^2+^]_i_ were displayed as F_t_/F_0_— a ratio of real-time fluorescence (F_t_) relative to the intensity at the beginning (F_0_) of the experiment. Maximal change in [Ca^2+^]_i_ was presented as ratio F_1_/F_0_. All experiments were performed at room temperature (~23 °C). To inhibit TRPC5, cells were pretreated with T5E3 (15 μg ml^−1^) or pre-immune serum IgG (15 μg ml^−1^) for 45 min. Culture neurons were transiently transfected with T5DN or empty vector using electroporation if needed, and were used for experiments 48–72 h afterward.
